# Local proliferation initiates macrophage accumulation in adipose tissue during obesity

**DOI:** 10.1038/cddis.2016.54

**Published:** 2016-03-31

**Authors:** C Zheng, Q Yang, J Cao, N Xie, K Liu, P Shou, F Qian, Y Wang, Y Shi

**Affiliations:** 1 Key Laboratory of Stem Cell Biology, Institute of Health Sciences, Shanghai Institutes for Biological Sciences, Chinese Academy of Sciences/Shanghai Jiao Tong University School of Medicine, Shanghai, China; 2 Biochemistry IDI-IRCCS Laboratory, Department of Experimental Medicine and Surgery, University of Rom ‘Tor Vergata', Rome, Italy; 3 School of Life Science and Technology, ShanghaiTech University, Shanghai, China; 4 The First Affiliated Hospital of Soochow University, Institutes for Translational Medicine, Soochow University, Suzhou, China; 5 Child Health Institute of New Jersey, Rutgers-Robert Wood Johnson Medical School, New Brunswick, NJ, USA

## Abstract

Obesity-associated chronic inflammation is characterized by an accumulation of adipose tissue macrophages (ATMs). It is generally believed that those macrophages are derived from peripheral blood monocytes. However, recent studies suggest that local proliferation of macrophages is responsible for ATM accumulation. In the present study, we revealed that both migration and proliferation contribute to ATM accumulation during obesity development. We show that there is a significant increase in ATMs at the early stage of obesity, which is largely due to an enhanced *in situ* macrophage proliferation. This result was obtained by employing fat-shielded irradiation and bone marrow reconstitution. Additionally, the production of CCL2, a pivotal chemoattractant of monocytes, was not found to be increased at this stage, corroborating with a critical role of proliferation. Nonetheless, as obesity proceeds, the role of monocyte migration into adipose tissue becomes more significant and those new immigrants further proliferate locally. These proliferating ATMs mainly reside in crown-like structures formed by macrophages surrounding dead adipocytes. We further showed that IL-4/STAT6 is a driving force for ATM proliferation. Therefore, we demonstrated that local proliferation of resident macrophages contributes to ATM accumulation during obesity development and has a key role in obesity-associated inflammation.

The accumulation of adipose tissue macrophages (ATMs) is a significant characteristic of obesity-associated chronic inflammation. It is also critical in regulating obesity development. In lean animals, there is a low cellularity of resident ATMs interspersing among adipocytes, which are considered as M2 macrophages. During obesity, significantly increased macrophages accumulate in adipose tissue and form the so-called ‘crown-like structures' (CLSs) around the dead adipocytes.^1, 2^ Those macrophages exhibit M1 phenotype and produce various types of inflammatory cytokines, such as TNF-*α*, resulting in the propagation of obesity-related inflammation and the development of metabolic disorders, such as insulin resistance.^3, 4, 5^

Traditionally, the accumulated ATMs are considered as a consequence of peripheral monocyte migration under inflammatory conditions. Recently, increasing evidences have shown that the maintenance of tissue macrophages is probably independent of the replenishment of circulating monocytes and even independent of precursors from bone marrow.^6^ Indeed, several kinds of tissue macrophages are capable of self-renewal and proliferate locally in naive state, such as microglia,^7, 8^ Kupffer cells,^9^ and Langerhans cells.^10^In acute inflammation status, for instance, during parasitic infection, local proliferation of macrophages is boosted and these macrophages exhibit phenotypes of alternatively activated macrophages, a process driven by Th2 cytokines.^11^ In chronic inflammation conditions, such as atherosclerosis, local proliferation of macrophages also occurs and contributes to macrophage accumulation in arterial walls.^12^ Most recently, it has been reported that local proliferation of macrophages could contribute to the ATM accumulation in obesity.^13, 14^

Given the potential contributions of monocyte migration and macrophage proliferation to ATM accumulation, an important question about the respective role of each event in ATM accumulation during obesity is raising. To address it, we first focus on the initiation of ATM accumulation in obesity. We found that, although there is no significant change in the level of chemokine (C-C motif) ligand 2 (CCL2) either in adipose tissue or in circulation, the cellularity of ATMs is dramatically elevated at the early stage of obesity. Interestingly, the increase of ATMs was accompanied with vigorous ATM proliferation. By inducing obesity in chimeric mice that were generated by fat-shielded irradiation and bone marrow transplantation, we demonstrated that *in situ* proliferation of resident macrophages dominates the initiation of ATM accumulation at early stage of obesity, and the recruited monocytes make contribution to ATM accumulation at a relatively late stage of obesity. This study sheds light on the dynamic process of ATM accumulation and provides insight on the initiation of obesity-associated inflammation.

## Results

### ATM accumulation at the early stage of obesity is related to macrophage proliferation

**Figure 1:**
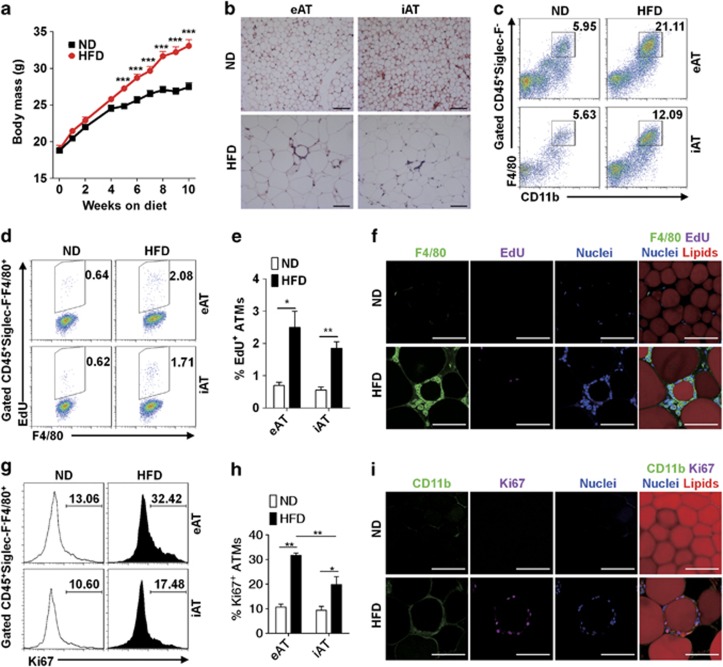
Macrophage accumulation in adipose tissue is associated with *in situ* proliferation at the early stage of obesity. (**a**) Body weight of mice fed with ND and HFD (*n*=10 mice in each group). Error bars represent means±S.E.M. ***P<0.001. (**b**) Hematoxylin and eosin staining showing leukocyte infiltration in eAT and iAT. Scale bars, 100 *μ*M. (**c**) Flow cytometric analysis of ATM accumulation in eAT and iAT from ND- or HFD-treated mice. Representative dot plots are shown. Numbers on the graphs indicate the percentage of gated events in total events recorded. (**d**) Flow cytometric analysis of EdU incorporation in ATMs from eAT and iAT of mice on ND or HFD. Mice were pulsed with 10 *μ*g EdU/g body weight for 3 h. (**e**) Summarizing of the data in panel (**d**) are shown as means±S.E.M. *n*=3–4 mice in each group, *P<0.05, **P<0.01. (**f**) Immunofluorescence staining for EdU-incorporated ATMs in eAT of mice on ND or HFD. Scale bars, 100 *μ*m. (**g**) Flow cytometric analysis of Ki67 expression in ATMs from eAT and iAT of mice on ND or HFD. (**h**) Summarizing of the data in panel (**g**) are shown as means±S.E.M. *n*=3–4 mice in each group, *P<0.05, **P<0.01. (**i**) Immunofluorescence staining for Ki67^+^ ATMs in eAT of mice on ND or HFD. Scale bars, 100 *μ*m

Macrophage accumulation in adipose tissue is a significant characteristic of obesity and promotes the chronic inflammation. It has been demonstrated that macrophage accumulation in inflamed tissue can be caused by both monocyte recruitment and macrophage local proliferation. However, the contributions of these two processes on macrophage accumulation in adipose tissue during obesity remain unclear. To address this question, we performed diet-induced obesity model by feeding C57BL/6 mice with high-fat diet (HFD) (Figure 1a). Eight weeks later, a relatively early stage of obesity, epididymal adipose tissue (eAT) and inguinal adipose tissue (iAT) were isolated and analyzed histologically and flow cytometrically. Significant leukocyte accumulation was observed in adipose tissue, forming CLSs (Figure 1b). We further analyzed immune cell populations in adipose tissue and found that the percentage of ATMs (CD45^+^Siglec-F^−^CD11b^+^F4/80^+^) is dramatically increased in both eAT and iAT from mice fed with HFD for 8 weeks, comparing with that from normal diet (ND)-treated mice (Figure 1c). Next we examine whether *in situ* macrophage proliferation is related to ATM accumulation at the early stage of obesity. We performed EdU (5-ethynyl-2′-deoxyuridine) incorporation assay, which specifically identifies the proliferating cells in S phase of cell cycle. Mice fed with an ND or a HFD for 8 weeks were pulsed with EdU for 3 h, and then eAT and iAT were isolated and analyzed for macrophages with EdU incorporation by flow cytometry. Notably, the EdU-incorporated ATMs in both eAT and iAT of HFD-treated mice were greatly increased than that of ND-treated mice (Figures 1d and e and Supplementary Figure S1a). It is worth noting that there was no detectable EdU-incorporated monocyte in blood (data not shown), excluding the possibility that EdU-incorporated macrophages in adipose tissue were from circulation. To verify whether the ATMs indeed proliferate *in situ*, we performed whole-mount staining on eAT and found a significant increase in EdU-incorporated macrophages in adipose tissue of obese mice but not in that of lean mice. Interestingly, these EdU-incorporated macrophages primarily resided in the CLSs formed by the accumulated ATMs (Figure 1f). Consistently, the Ki67-positive ATMs in eAT and iAT derived from HFD mice were significantly increased as compared with that of ND-treated mice (Figures 1g and h). Interestingly, we found that, even in the ND-treated mice, there was a basal level of Ki67-expressing ATMs, indicating that proliferation is also involved in the maintenance of the population of ATMs under lean status. Additionally, consistent with a much higher level of ATM accumulation in eAT than in iAT, the Ki67^+^ ATMs in eAT was significantly higher than that of iAT in HFD-treated mice (Figures 1c, g and h). By detecting *in situ* Ki67 expression in ATMs, we found that the Ki67^+^ ATMs are dramatically increased in eAT of HFD-treated mice than that of ND-treated mice, preferentially localizing in the CLSs (Figure 1i). These results demonstrate that *in situ* proliferation of macrophages occurs at the early stage of obesity and contributes to ATM accumulation.

### The early stage of genetic obesity is accompanied with ATM proliferation

**Figure 2:**
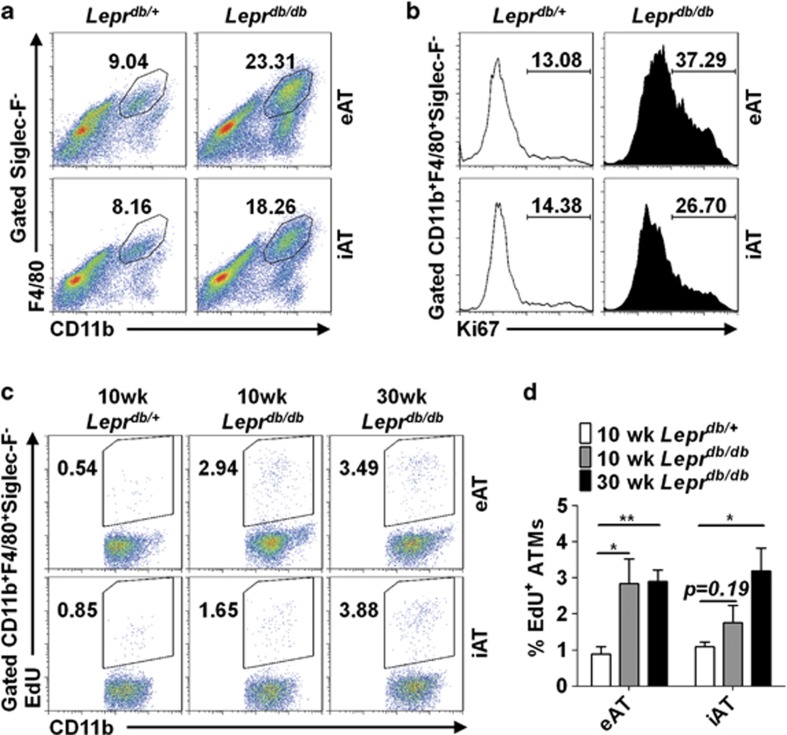
Proliferation of ATMs at the early stage of genetic obesity. (**a**) Flow cytometric analysis of ATM accumulation in eAT and iAT from 7-week-old leptin receptor-deficient mice (*Lepr*^*db/db*^) and their heterozygous littermates (*Lepr*^*db/+*^). Representative dot plots are shown. Numbers on the graphs indicate the percentage of gated events in total events recorded. (**b**) Flow cytometric analysis of Ki67 expression in ATMs from eAT and iAT of 7-week-old leptin receptor-deficient mice (*Lepr*^*db/db*^) and heterozygous littermates (*Lepr*^*db/+*^). (**c**) Flow cytometric analysis of EdU incorporation in ATMs from eAT and iAT of *Lepr*^*db/db*^ mice and their heterozygous siblings. (**d**) Summarizing of the data in panel (**c**) are shown as means±S.E.M. *n*=3–4 mice in each group, *P<0.05, **P<0.01

Obesity is a consequence of complex gene–environment interactions. In addition to diet-induced obesity, we also investigated whether *in situ* proliferation of macrophages is involved in ATM accumulation in one model of the genetically inherited obesity, the leptin receptor-deficient mice (*Lepr*^*db/db*^ mice, commonly referred to as *db/db* mice). We found that the ATM accumulation in adipose tissue (eAT and iAT) of *Lepr*^*db/db*^ mice is dramatically increased at the initial stage of obesity (7-week old) as compared with that of their heterozygous littermates (Figure 2a). Meanwhile, Ki67^+^ macrophages were greatly increased in adipose tissue of 7-week-old *Lepr*^*db/db*^ mice (Figure 2b). We further performed EdU incorporation assay and found that, at the early stage of genetic obesity (10-week old), significantly more EdU-incorporated macrophages were observed in adipose tissue of *Lepr*^*db/db*^ mice (Figures 2c and d). The percentage of EdU-incorporated ATMs from mice in advanced genetic obesity (30-week old) was also significantly higher than that of lean littermates. Thus macrophage proliferation contributes to ATM accumulation in genetic obesity.

### ATM accumulation in obesity is dominated by *in situ* proliferation

**Figure 3:**
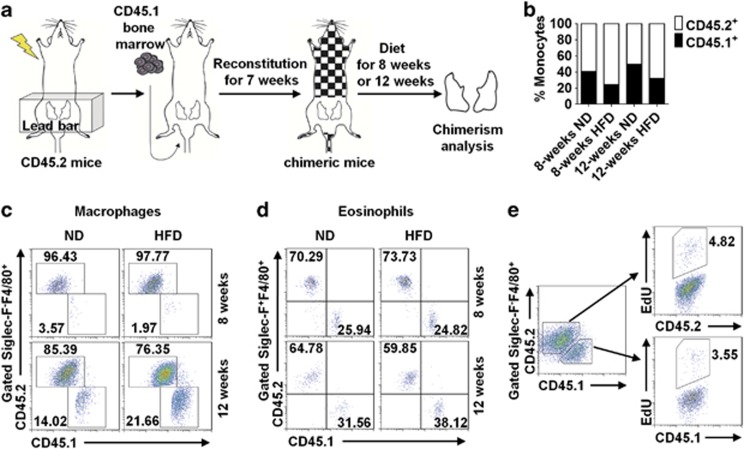
*In situ* proliferation dominates the initiation of ATM accumulation during obesity. (**a**) Schematic representation of fat-shielded irradiation and bone marrow transplantation. CD45.2 mice were shielded with lead above the abdomen before lethal irradiation, transplanted with CD45.1 bone marrow, left for reconstitution for 7 weeks, and subjected to diet treatment for 8–12 weeks. Chimerism analysis was performed on leukocytes in blood and eAT. (**b**) Percentage of chimerism in blood monocytes (CD11b^+^CD115^+^) from chimeric mice subjected to ND or HFD for either 8 or 12 weeks. (**c**) Contribution of CD45.1 donor-derived macrophages to ATM accumulation was analyzed on chimeric mice fed with ND or HFD for 8 or 12 weeks. (**d**) Contribution of CD45.1 donor-derived eosinophils to adipose tissue eosinophils was analyzed on chimeric mice subjected to ND or HFD for 8 or 12 weeks. The representative results shown in panels (**b**, **c**, and **d**) were from the same group of chimeric mice. (**e**) Flow cytometric analysis of EdU incorporation in ATMs from chimeric mice on HFD for 12 weeks

To determine the respective contributions of macrophage local proliferation and monocyte migration to ATM accumulation, we generated bone marrow chimeric mice with a modified strategy. In this experiment, the lower abdomens of C57BL/6 mice (with CD45.2 allotype) were shielded during lethal irradiation to protect the resident ATMs in eAT from genotoxic insult. These mice were then transplanted with bone marrow cells from CD45.1 congenic mice. Seven weeks later, these mice were fed with HFD (Figure 3a). To examine whether the chimeric mice were successfully generated, we analyzed blood monocytes of recipient mice under different treatments and found that the percentage of CD45.1^+^ blood monocytes in periphery blood was around 30% (Figure 3b), indicating an efficient engraftment of donor bone marrow cells. We then examined the chimeric level of donor-derived ATMs in adipose tissue. After feeding mice with HFD for 8 weeks, despite an increase in total macrophages in eAT, the percentage of donor-derived (CD45.1^+^) ATMs remained at an extremely low level (2%), similar to those in ND-treated mice (3.6%) (Figure 3c). These data indicate that proliferation of resident macrophages rather than migration of blood monocytes dominates the initiation of ATM accumulation. It is of interest that, with HFD feeding for 12 weeks, an obvious increase in the percentage of donor-derived (CD45.1^+^) macrophages in adipose tissue of recipient mice was observed, suggesting that the monocytes migration makes contribution to accumulation of ATMs at late stage of obesity (Figure 3c).

As a comparison, we further analyzed the chimeric level of eosinophils in adipose tissue. Eosinophils are another subset of myeloid cells that have been shown to migrate from peripheral blood to adipose tissue and have an important role in sustaining the glucose homeostasis,^15^ which do not proliferate either in obese mice or in lean mice (Supplementary Figures S1a and b). Different from ATMs, obvious donor-derived (CD45.1^+^) eosinophils were observed in adipose tissue of obese mice, at both the early stage (8-week HFD) and the late stage of obesity (12-week HFD) (Figure 3d). Therefore, we verified that ATM accumulation is initiated by resident macrophage proliferation at the early stage of obesity and further promoted by monocyte migration at the late stage of obesity.

We further raised an intriguing question: is the proliferation potency of macrophages restricted to the resident ATMs or could it also be observed in recruited macrophages? To address it, we performed EdU incorporation assay on the chimeric mice fed with HFD for 12 weeks. Interestingly, both CD45.2^+^ and CD45.1^+^ EdU-incorporated ATMs were detected in adipose tissue (Figure 3e), indicating that proliferation of macrophage occurs regardless of their origins. Thus local proliferation of both resident and recruited macrophages contributes to ATM accumulation.

### CCL2 is dispensable for macrophage accumulation at the early stage of obesity

**Figure 4:**
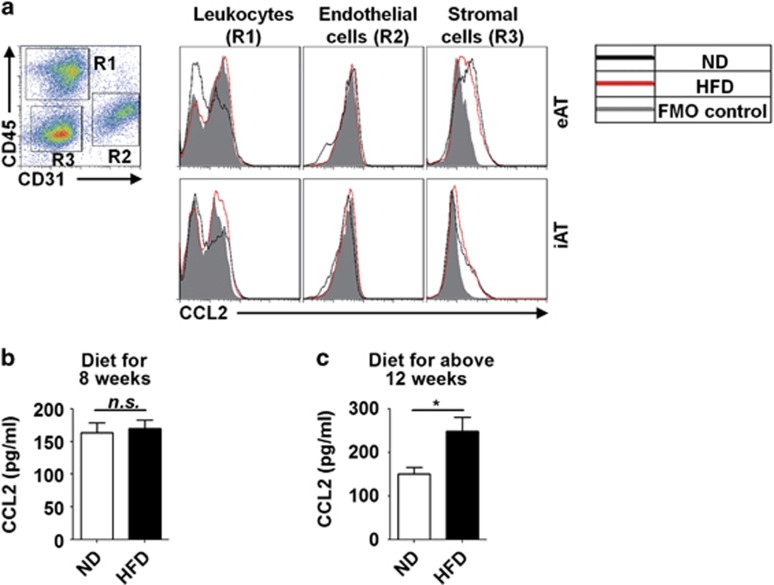
The level of CCL2 in adipose tissue and serum. (**a**) Flow cytometric analysis of CCL2 expression in leukocytes, endothelial cells, and stromal cells in eAT and iAT from ND- or HFD-treated mice. Representative dot plots are shown. CD45^+^ cells (R1) represent leukocytes, CD31^+^ cells (R2) represent endothelial cells, and CD45^−^ CD31^−^ cells (R3) represent stromal cells. (**b** and **c**) CCL2 expression level in serum of mice at early-stage (treated with diet for 8 weeks) (**b**) or relatively late-stage (treated with diet for >12 weeks) of obesity (**c**). Means±S.E.M. are shown. *n*=5–8 mice in each group, *P<0.05

To reinforce the conclusion that proliferation rather than monocyte migration plays critical role in ATM accumulation at the early stage of obesity, we analyzed the local expression of CCL2 (MCP-1) in adipose tissue, which is the pivotal chemoattractant for monocyte migration.^16^ Interestingly, employing intracellular staining, we found that CCL2 was mainly expressed by CD45^−^CD31^−^ stromal cells in adipose tissue rather than CD45^+^ leukocytes or CD31^+^ endothelial cells. In fact, no enhanced CCL2 expression was observed in any of the three populations of cells derived from adipose tissue of obese mice as compared with that of lean mice (Figure 4a). Consistently, the level of CCL2 in serum was not changed at the early stage of obesity (Figure 4b). However, at a later stage of obesity (HFD for >12 weeks), a significant higher level of CCL2 in the serum was found (Figure 4c). These results indicate that CCL2-induced monocyte migration is not the determinant factor for ATM accumulation at the early stage of obesity, whereas it may regulate ATM accumulation at a relatively later stage of obesity.

### ATMs with mitogenic activity are localized in CLSs

**Figure 5:**
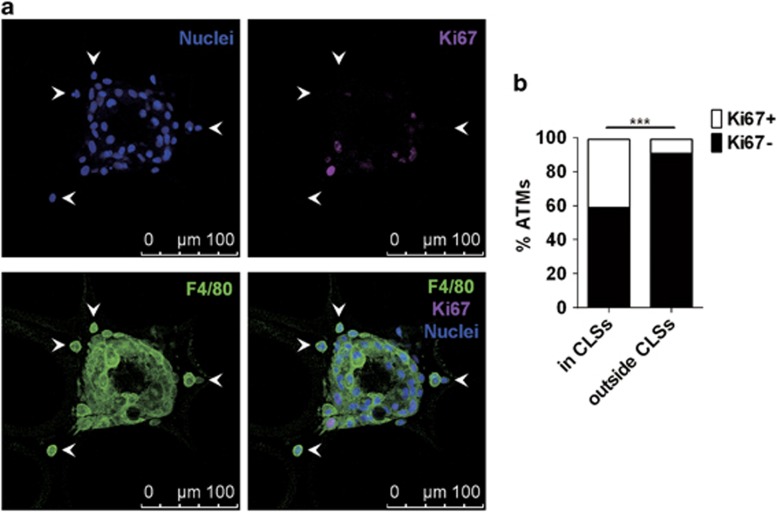
Proliferating ATMs preferentially reside in CLSs. (**a**) Immunofluorescence showing the localization of Ki67^+^ ATMs in HFD-induced obese mice. Arrowheads indicate the ATMs outside CLSs. Scale bars, 100 *μ*m. (**b**) Percentage of Ki67^+^ ATMs in CLSs and outside CLSs, summarizing 225 ATMs from 7 immunofluorescence pictures. ***P<0.001, Fisher's exact test

We noticed that the majority of proliferating ATMs resided in the CLSs in adipose tissue of mice at the early stage of obesity (Figures 1f and i). In these structures, adipocytes rimmed by immune cells are regarded to be necrotic or apoptotic.^17, 18^ We further found that Ki67^+^ ATMs were preferentially localized in the CLSs of obese mice at the advanced stage, and ATMs outside of these structures scarcely expressed Ki67 (Figure 5a). By statistically analyzing Ki67-expressing ATMs in mice at different stages of obesity, we found that the percentage of Ki67^+^ ATMs in CLSs was dramatically higher than that outside CLSs (Figure 5b). Of note, the percentage of Ki67^+^ ATMs outside CLSs (about 10%) in obese mice was comparable to that of lean mice (Figures 1g and h), indicating that these ‘free' ATMs only maintain a basal level of self-renewal as ATMs do in lean mice. Thus proliferating ATMs preferentially localize in the CLSs, suggesting that the mitogenic stimuli for ATM proliferation in obesity could relate to these structures.

Macrophages are important regulators of inflammation in adipose tissue by producing various types of cytokines. Among them, TNF-*α* is a major proinflammatory cytokine of classically activated macrophages, and Resistin-like molecule alpha (RELM-*α*) is a typical cytokine produced by alternatively activated macrophages. To verify the expression of these cytokines by proliferating ATMs, we performed intracellular staining. We found that the percentage of RELM-*α*^+^ ATMs was gradually decreased along with the progression of obesity. Detailed analysis showed that the expression of RELM-*α* is much lower in the Ki67^+^ ATMs as compared with that in the Ki67^−^ ATMs (Supplementary Figure S2). TNF-*α*^+^ ATMs were increased at first and then decreased during the progression of obesity. The percentage of Ki67^+^TNF-*α*^+^ ATMs are comparable to that of Ki67^−^TNF-*α*^+^ ATMs (Supplementary Figure S3).

### IL-4/STAT6 signaling is the major driving force for ATM proliferation

**Figure 6:**
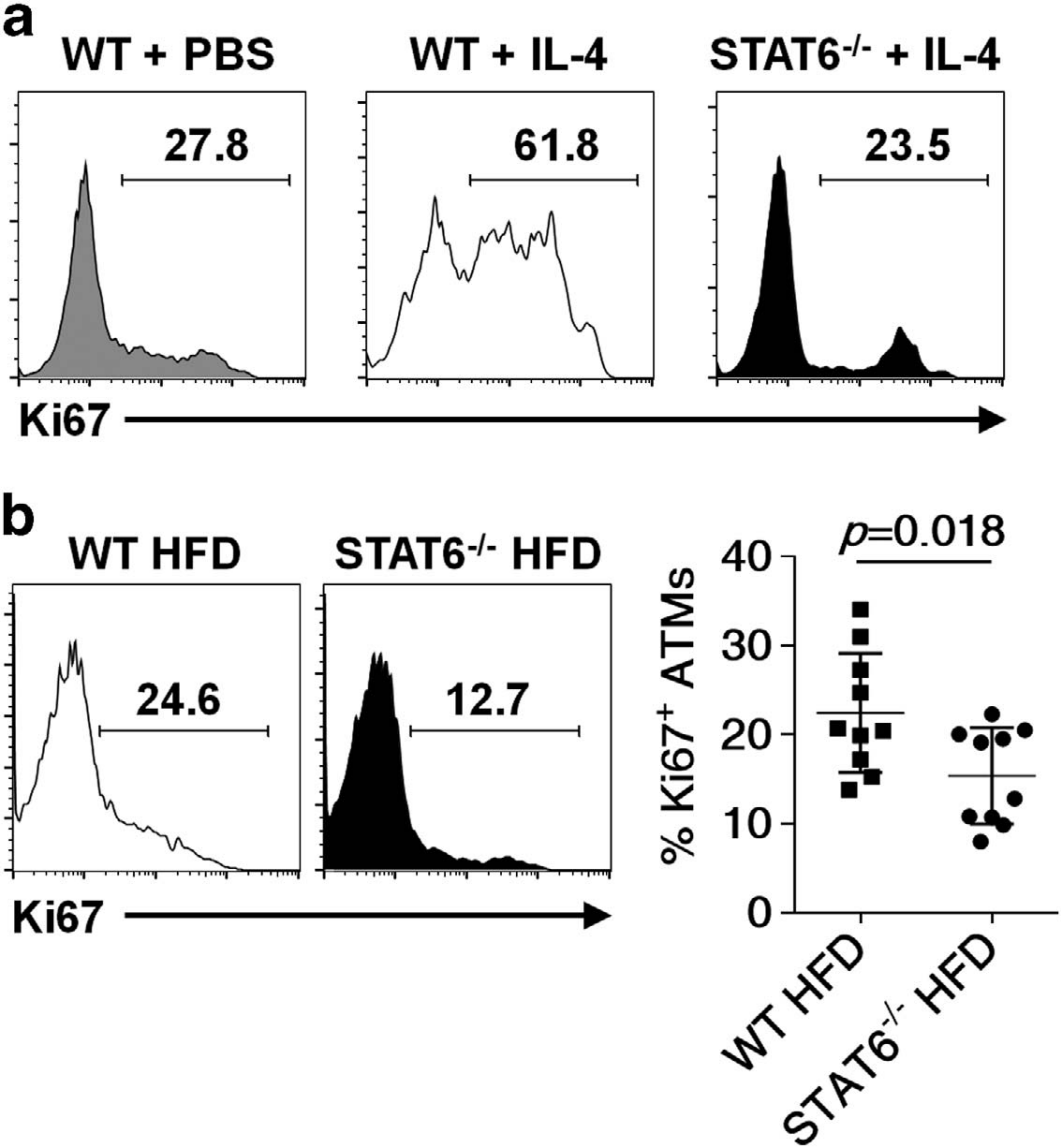
IL-4/STAT6 drives the proliferation of ATMs. (**a**) Ki67 expression in ATMs from eAT of WT and STAT6^−/−^ mice treated with a complex of IL-4 and IL-4 antibody (to prolong the bioavailability of IL-4 *in vivo*) or PBS for 48 h. (**b**) Ki67 expression in ATMs from eAT of WT and STAT6^−/−^ mice on HFD (*n*=10 in each group). Means±S.E.M. are shown

We further investigated the mitogenic stimuli for ATM proliferation. It has been shown that IL-4,^11^ M-CSF,^12^ and CCL2^13^ could drive the local proliferation of macrophages. However, when we examined the effects of these cytokines on ATM proliferation, we found that only the administration of IL-4 could boost the ATM proliferation in lean mice.^19^ When lean mice were intraperitoneally injected with IL-4, a dramatic increase of Ki67^+^ ATMs was observed (Figure 6a). After binding to its receptor, IL-4 activates JAK1 and JAK3 and subsequently results in the phosphorylation of STAT6, a central transcription factor for IL-4-mediated biological responses.^20^ We found that the proliferation of ATMs induced by IL-4 was greatly impaired in STAT6-deficient mice (Figure 6a), suggesting a critical role of IL-4/STAT6 signaling in the IL-4-driven ATM proliferation. More importantly, when mice were on HFD, the percentage of Ki67^+^ ATMs in STAT6-deficient mice was significantly lower than that of WT mice (Figure 6b). Thus the IL-4/STAT6 signaling is the driving force for ATM proliferation in obesity.

## Discussion

**Figure 7:**
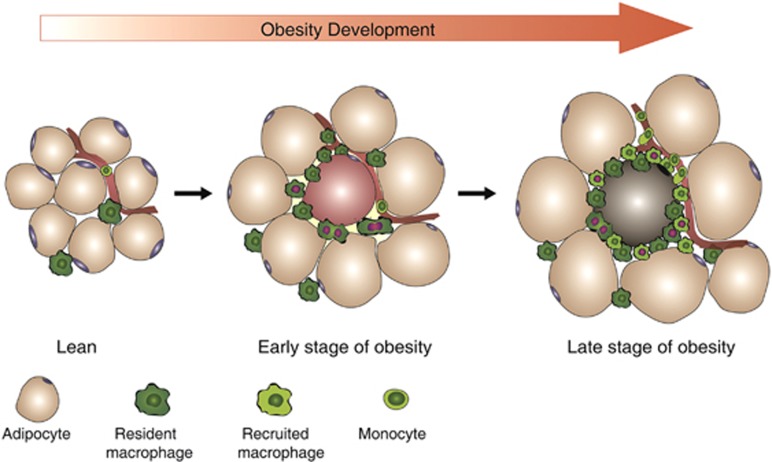
Schematic depiction of contribution of macrophage proliferation and monocyte recruitment to ATM accumulation in obesity. In lean mice, resident macrophages in adipose tissue are in quiescent status, maintaining a basal level of cellularity by self-renewal. At the early stage of obesity, these resident macrophages proliferate and lead to ATM accumulation, which is driven by stimuli released from adipocytes or/and other immune cells. At the late stage of obesity, ATM accumulation is further augmented by monocyte-derived macrophages, which also exhibit the proliferative capability. These proliferating macrophages always localize in the CLSs and surround the apoptotic adipocyte. Red nuclei indicate that the macrophages are proliferating

Obesity is associated with accumulation of ATMs, which propagates chronic inflammation and promotes insulin resistance. Previous reports have showed that the migration of blood monocytes contributes to macrophage infiltration in inflamed sites.^1, 16, 21^ Recent evidences indicate that local proliferation is also involved in regulating ATM accumulation in obesity.^13, 14^ However, the respective contributions of these two events to ATM accumulation during obesity progression have not been addressed. In this study, we demonstrated that *in situ* proliferation of resident macrophages determines ATM accumulation at the early stage of obesity, whereas immigrated monocytes contribute to ATM accumulation at a relatively late stage of obesity. Furthermore, both resident and recruited macrophages proliferate in the adipose tissue of obese mice (summarized in Figure 7).

Previously, it has been reported that the infiltration of peripheral monocytes exerts an important role in ATM accumulation in obesity.^1^ As one of the most pivotal chemokines in mediating monocyte recruitment, CCL2 (MCP-1) has been suggested to mediate the accumulation of ATMs during obesity.^16^ However, other report shows that, in the absence of CCL2, no reduction in macrophage accumulation in adipose tissue was observed.^22^ These controversial results suggest that migration of monocytes could not be the unique pathway in regulating macrophage accumulation in adipose tissue during obesity development.

In fact, resident macrophages in various types of tissues, such as lung, splenic red pulp, peritoneum, and bone marrow, could maintain their population by local proliferation in the quiescent state, with little replenishment from circulating monocytes and hematopoietic progenitors.^23^ In addition to quiescent state, during parasite infection, IL-4-induced local proliferation determines the accumulation of macrophages in pleural cavity.^11^ Furthermore, during chronic inflammatory response, such as in atherosclerosis, macrophage accumulation in lesion is mainly dominated by local proliferation driven by M-CSF.^12^ Consistently, we found that, even in lean mice, a low level of ATM proliferation can be observed, representing a basal self-renewal of resident ATMs under naive status. Strikingly, the proliferation rate of ATMs was greatly enhanced after mice were fed with HFD for 8 weeks. However, this was not related to the enhanced levels of CCL2 in serum and adipose tissue of obese mice. We further hypothesize that *in situ* proliferation of resident macrophages could be the driving force for the initiation of ATM accumulation. We tested this hypothesis by generating chimeric mice with protection on their adipose tissue during lethal irradiation and with bone marrow transplantation. Feeding these mice with HFD, we found that the increased ATMs at the early stage of obesity are mainly derived from recipients, rather than donors. However, at the late stage of obesity, a significant increase of the donor-derived ATMs was found, accompanying with enhanced CCL2 level in the serum. Thereby, ATM accumulation in obesity is mainly resulting from local proliferation of resident macrophages at the early stage and contributed by monocyte replenishment at the late stage. These findings have important clinical implications for intervention of obesity-related insulin resistance. Strategies to suppress the CCL2/CCR2 signal may only partially inhibit ATM accumulation and insulin resistance at the late stage of obesity. However, targeting the stimuli or its related signaling pathway for ATM proliferation could guide the development of promising intervention for obesity-related inflammation and insulin resistance.

Our findings show that resident macrophages are enriched in adipose tissue of mice during the lean and early obesity phases. By analyzing the phenotype of ATMs during the progression of obesity, we found that, during the lean and early obesity phases, there is a substantial number of alternatively activated macrophages; however, at the late obesity stage, this type of macrophages is dramatically reduced. Interestingly, it has been reported that alternatively activated macrophages have enhanced capacity of lipid catabolism.^24, 25, 26^ Thus alternatively activated macrophages at the early stages of obesity are the major population of resident macrophages, with more specialized lipid catabolism than that of immigrant macrophages.

We found that ATMs in cell cycle progression mainly reside in CLSs in adipose tissue, whereas ATMs outside these structures exhibit much less proliferation capacity, similar to that of ATMs in lean mice. These results suggest that the potential molecules in CLS microenvironment may provide the mitogenic stimuli for obesity-related ATM proliferation. These stimuli could be produced by the apoptotic or necrotic adipocytes in the core of CLSs or provided by the nearby immune cells, such as ATMs and eosinophils. Recently, it has been reported that CCL2 is associated with the local proliferation of ATMs.^13^ Also, M-CSF is regarded as a possible candidate for regulating the mitogenic activity of macrophages.^23^ We found that neither CCL2 nor M-CSF administration promoted the ATM proliferation in lean mice. However, IL-4 administration greatly enhanced the proliferation of ATMs in lean mice. Importantly, a significant reduction in the *in situ* proliferation of ATMs was observed in the HFD-treated STAT6-deficient mice as compared with that of wild-type mice, demonstrating an important role of IL-4 in driving the local proliferation of ATMs in obesity.

In conclusion, we found that macrophage accumulation in adipose tissue during obesity is initiated by *in situ* proliferation of resident ATMs and further promoted by a concerted activation of monocyte migration and macrophage proliferation. Our observations provide insight on the development of obesity-related inflammation and could guide the designing of therapeutic strategies for obesity-associated disorders.

## Materials and Methods

### Animal experiments

C57BL/6 mice were purchased from the Shanghai Laboratory Animal Center of Chinese Academy of Sciences (Shanghai, China). CD45.1 mice in C57BL/6 background were kindly provided by Dr. Yanyun Zhang of the Institute of Health Sciences of the Chinese Academy of Sciences. Heterozygous leptin receptor-deficient mice (*Lepr*^*db/+*^) in C57BL/6 background were purchased from Model Animal Research Center of Nanjing University (Nanjing, China) and were inbred in our laboratory. STAT6^−/−^ (C.129S2-Stat6^tm1Gru^/J) mice were from Jackson Laboratory (Bar Harbor, ME, USA). All experiments were approved by the Institutional Animal Care and Use Committee of the Institute of Health Sciences, Shanghai Institutes for Biological Sciences of Chinese Academy of Sciences.

Diet-induced obesity was performed by feeding 5-week-old mice with a HFD containing 60 kcal% fat (D12492, Research Diets, New Brunswick, NJ, USA). The control group was fed with a ND. EdU incorporation assay was performed with the Click-iT EdU Assay Kits (Life Technologies, Carlsbad, CA, USA) according to the manufacturer's instruction. Mice were i.p. injected with 10 *μ*g EdU/g body weight 3 h prior to killing. The Click-iT reaction was carried out on adipose tissue and analyzed flow cytometrically and immunohistologically. To assess the contribution of resident macrophages to ATMs accumulation, the lower abdomen of anesthetized CD45.2 mice was shielded with a 6-cm-thick lead block. Those mice were exposed to a dose of 9.5 Gy *γ* irradiation and i.v. injected with 10^7^ CD45.1 bone marrow cells. Chimeric mice were left for 7 weeks for reconstitution prior to diet treatment. To examine the role of IL-4 in ATM proliferation, every mouse was i.p. injected with a combination of 5 *μ*g IL-4 (Peprotech, Rocky Hill, NJ, USA) and 25 *μ*g IL-4 antibody (clone 11B11, Harlan Bioproducts for Science, Indianapolis, IN, USA), or PBS, and ATM analysis was performed 48 h later.

### Flow cytometry

The eAT and iAT were collected from killed mice, cut into small pieces, and rinsed with PBS before digestion with 2 mg/ml collagenase I (Sigma-Aldrich, St. Louis, MO, USA) for 1.5 h. Floating adipocytes were separated by centrifuging the cell suspension at 600 × *g* for 10 min. The pallets were re-suspended, filtered through 70-*μ*m sieves, and subjected to erythrocyte lysis. The cell suspension was preincubated with anti-CD16/CD32 (eBioscience, San Diego, CA, USA) to block Fc receptors before surface molecule staining. Intracellular staining was performed with fixation and permeabilization kits according to the manufacturer's instructions (eBioscience). The rat monoclonal antibodies, including F4/80, CD45, CD11b, CD45.1, CD45.2, Ki67, and TNF-*α*, were from eBioscience; CD115 and Siglec-F were from Biolegend (San Diego, CA, USA).

For intracellular staining of RELM-*α* and TNF-*α* in ATMs, 1 : 1000 GolgiPlug (BD Biosciences, San Jose, CA, USA) were added to collagenase during tissue digestion. Following surface molecules staining, intracellular staining was performed using a fixation kit and a permeabilization kit according to the manufacturer's instructions (eBioscience). Rabbit anti-RELM-*α* (Abcam, Cambridge, MA, USA), and Alexa Fluor 488-conjugated goat anti-rabbit IgG (Life Technologies) were used.

### Immunohistochemistry

The adipose tissue was cut into small pieces and fixed with 4% paraformaldehyde for 24 h at 4 °C. Whole-mount staining was then performed. Briefly, the specimens were permeabilized with 1% Triton X-100, blocked with 1% BSA and 3% FBS in PBS, and then incubated with respective antibodies for the surface or nuclear molecules. The adipocytes were counterstained with BODIPY 558/568 C12 (Invitrogen) in some experiments.

### Statistical analysis

Data are presented as means±S.E.M., and the significance was assessed by unpaired two-tailed *t*-test unless otherwise indicated. We considered *P*<0.05 as statistically significant.

## Abbreviations

ATM: adipose tissue macrophage
eAT: epididymal adipose tissue
iAT: inguinal adipose tissue
CCL2: chemokine (C-C motif) ligand 2
HFD: high-fat diet
ND: normal diet
EdU: 5-ethynyl-2′-deoxyuridine
CLS: crown-like structure

## References

[bib1] Weisberg SP, McCann D, Desai M, Rosenbaum M, Leibel RL, Ferrante AW Jr. Obesity is associated with macrophage accumulation in adipose tissue. J Clin Invest 2003; 112: 1796–1808. 14679176 10.1172/JCI19246 PMC296995

[bib2] Xu H, Barnes GT, Yang Q, Tan G, Yang D, Chou CJ et al. Chronic inflammation in fat plays a crucial role in the development of obesity-related insulin resistance. J Clin Invest 2003; 112: 1821–1830. 14679177 10.1172/JCI19451 PMC296998

[bib3] Johnson AMF, Olefsky JM. The origins and drivers of insulin resistance. Cell 2013; 152: 673–684. 23415219 10.1016/j.cell.2013.01.041

[bib4] Hotamisligil GS, Shargill NS, Spiegelman BM. Adipose expression of tumor necrosis factor-alpha: direct role in obesity-linked insulin resistance. Science 1993; 259: 87–91. 7678183 10.1126/science.7678183

[bib5] Uysal KT, Wiesbrock SM, Marino MW, Hotamisligil GS. Protection from obesity-induced insulin resistance in mice lacking TNF-alpha function. Nature 1997; 389: 610–614. 9335502 10.1038/39335

[bib6] Schulz C, Perdiguero EG, Chorro L, Szabo-Rogers H, Cagnard N, Kierdorf K et al. A lineage of myeloid cells independent of Myb and hematopoietic stem cells. Science 2012; 336: 86–90. 22442384 10.1126/science.1219179

[bib7] Ajami B, Bennett JL, Krieger C, Tetzlaff W, Rossi FMV. Local self-renewal can sustain CNS microglia maintenance and function throughout adult life. Nat Neurosci 2007; 10: 1538–1543. 18026097 10.1038/nn2014

[bib8] Ginhoux F, Greter M, Leboeuf M, Nandi S, See P, Gokhan S et al. Fate mapping analysis reveals that adult microglia derive from primitive macrophages. Science 2010; 330: 841–845. 20966214 10.1126/science.1194637 PMC3719181

[bib9] Klein I, Cornejo JC, Polakos NK, John B, Wuensch SA, Topham DJ et al. Kupffer cell heterogeneity: functional properties of bone marrow-derived and sessile hepatic macrophages. Blood 2007; 110: 4077–4085. 17690256 10.1182/blood-2007-02-073841 PMC2190614

[bib10] Chorro L, Sarde A, Li M, Woollard KJ, Chambon P, Malissen B et al. Langerhans cell (LC) proliferation mediates neonatal development, homeostasis, and inflammation-associated expansion of the epidermal LC network. J Exp Med 2009; 206: 3089–3100. 19995948 10.1084/jem.20091586 PMC2806478

[bib11] Jenkins SJ, Ruckerl D, Cook PC, Jones LH, Finkelman FD, van Rooijen N et al. Local macrophage proliferation, rather than recruitment from the blood, is a signature of T(H)2 inflammation. Science 2011; 332: 1284–1288. 21566158 10.1126/science.1204351 PMC3128495

[bib12] Robbins CS, Hilgendorf I, Weber GF, Theurl I, Iwamoto Y, Figueiredo JL et al. Local proliferation dominates lesional macrophage accumulation in atherosclerosis. Nat Med 2013; 19: 1166–1172. 23933982 10.1038/nm.3258 PMC3769444

[bib13] Amano SU, Cohen JL, Vangala P, Tencerova M, Nicoloro SM, Yawe JC et al. Local proliferation of macrophages contributes to obesity-associated adipose tissue inflammation. Cell Metab 2014; 19: 162–171. 24374218 10.1016/j.cmet.2013.11.017 PMC3931314

[bib14] Haase J, Weyer U, Immig K, Kloting N, Bluher M, Eilers J et al. Local proliferation of macrophages in adipose tissue during obesity-induced inflammation. Diabetologia 2014; 57: 562–571. 24343232 10.1007/s00125-013-3139-y

[bib15] Wu D, Molofsky AB, Liang HE, Ricardo-Gonzalez RR, Jouihan HA, Bando JK et al. Eosinophils sustain adipose alternatively activated macrophages associated with glucose homeostasis. Science 2011; 332: 243–247. 21436399 10.1126/science.1201475 PMC3144160

[bib16] Kanda H, Tateya S, Tamori Y, Kotani K, Hiasa KI, Kitazawa R et al. MCP-1 contributes to macrophage infiltration into adipose tissue, insulin resistance, and hepatic steatosis in obesity. J Clin Invest 2006; 116: 1494–1505. 16691291 10.1172/JCI26498 PMC1459069

[bib17] Feuerer M, Herrero L, Cipolletta D, Naaz A, Wong J, Nayer A et al. Lean, but not obese, fat is enriched for a unique population of regulatory T cells that affect metabolic parameters. Nat Med 2009; 15: 930–U137. 19633656 10.1038/nm.2002 PMC3115752

[bib18] Cinti S, Mitchell G, Barbatelli G, Murano I, Ceresi E, Faloia E et al. Adipocyte death defines macrophage localization and function in adipose tissue of obese mice and humans. J Lipid Res 2005; 46: 2347–2355. 16150820 10.1194/jlr.M500294-JLR200

[bib19] Zheng CX, Yang Q, Xu CL, Shou PS, Cao JC, Jiang MH et al. CD11b regulates obesity-induced insulin resistance via limiting alternative activation and proliferation of adipose tissue macrophages. Proc Natl Acad Sci USA 2015; 112: E7239–E7248. 26669445 10.1073/pnas.1500396113 PMC4702980

[bib20] Kelly-Welch AE, Hanson EM, Boothby MR, Keegan AD. Interleukin-4 and interleukin-13 signaling connections maps. Science 2003; 300: 1527–1528. 12791978 10.1126/science.1085458

[bib21] Oh DY, Morinaga H, Talukdar S, Bae EJ, Olefsky JM. Increased macrophage migration into adipose tissue in obese mice. Diabetes 2012; 61: 346–354. 22190646 10.2337/db11-0860 PMC3266418

[bib22] Inouye KE, Shi H, Howard JK, Daly CH, Lord GM, Rollins BJ et al. Absence of CC chemokine ligand 2 does not limit obesity-associated infiltration of macrophages into adipose tissue. Diabetes 2007; 56: 2242–2250. 17473219 10.2337/db07-0425

[bib23] Hashimoto D, Chow A, Noizat C, Teo P, Beasley MB, Leboeuf M et al. Tissue-resident macrophages self-maintain locally throughout adult life with minimal contribution from circulating monocytes. Immunity 2013; 38: 792–804. 23601688 10.1016/j.immuni.2013.04.004 PMC3853406

[bib24] Odegaard JI, Ricardo-Gonzalez RR, Goforth MH, Morel CR, Subramanian V, Mukundan L et al. Macrophage-specific PPARgamma controls alternative activation and improves insulin resistance. Nature 2007; 447: 1116–1120. 17515919 10.1038/nature05894 PMC2587297

[bib25] Odegaard JI, Ricardo-Gonzalez RR, Red Eagle A, Vats D, Morel CR, Goforth MH et al. Alternative M2 activation of Kupffer cells by PPARdelta ameliorates obesity-induced insulin resistance. Cell Metab 2008; 7: 496–507. 18522831 10.1016/j.cmet.2008.04.003 PMC2587370

[bib26] Vats D, Mukundan L, Odegaard JI, Zhang L, Smith KL, Morel CR et al. Oxidative metabolism and PGC-1 beta attenuate macrophage-mediated inflammation. Cell Metab 2006; 4: 13–24. 16814729 10.1016/j.cmet.2006.05.011 PMC1904486

