# Supplementary material for: Local proliferation initiates macrophage accumulation in adipose tissue during obesity
Source: Cell Death Dis. 2016 Mar 31;7(3):e2167–. doi: 10.1038/cddis.2016.54 (PMC4823955; doi:10.1038/cddis.2016.54)
Supplement: Supplementary Information [file cddis201654x1.pdf]

Supplemental Figures

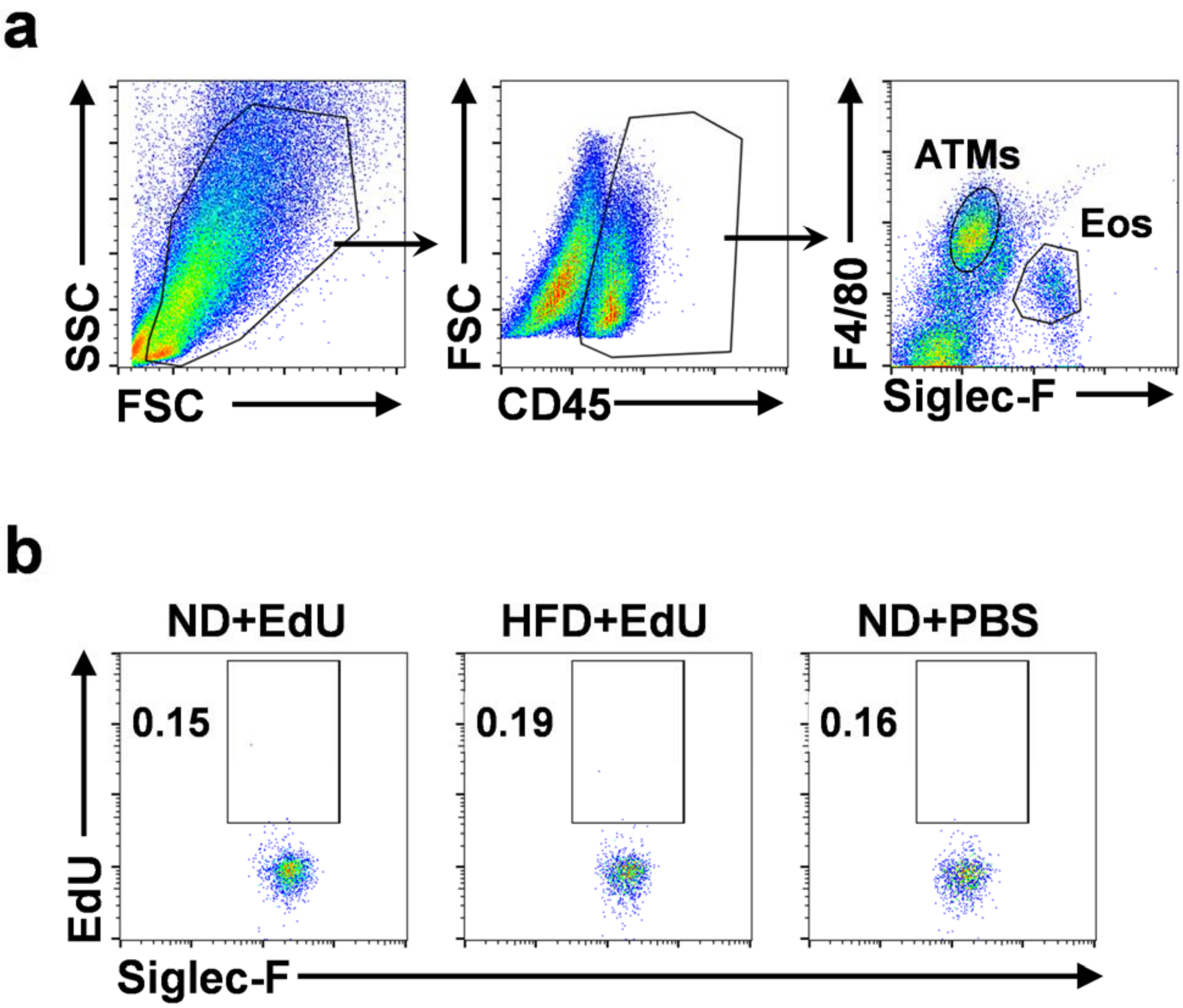

Figure S1. Eosinophils in adipose tissue do not proliferate. (a) Gating strategy of adipose tissue macrophages (ATMs) and eosinophils (Eos). ATMs are defined as CD45⁺F4/80⁺Siglec⁻F⁻, and eosinophils are defined as CD45⁺F4/80⁺Siglec-F⁺. (b) Flow cytometric analysis of EdU (5-ethynyl-2´-deoxyuridine) incorporation in eosinophils from epididymal adipose tissue of mice on ND or HFD. Mice were pulsed with 10 µg EdU/g body weight or PBS for 3 hours.

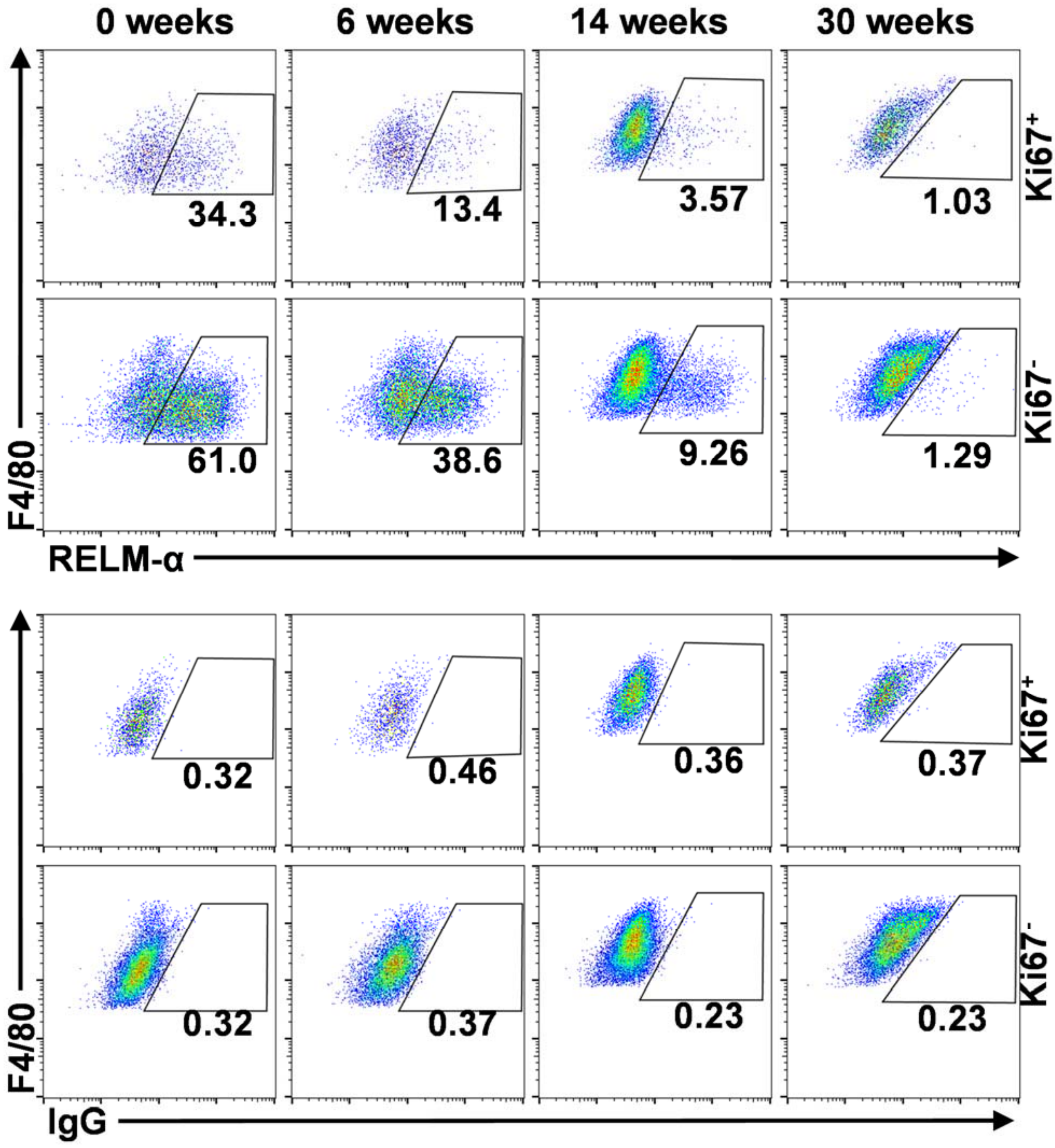

Figure S2. RELM-α expression in adipose tissue macrophages. Mice were treated with normal diet or high fat-diet for indicated time and epididymal adipose tissue were isolated for the analysis of RELM-α expression by intracellular staining.

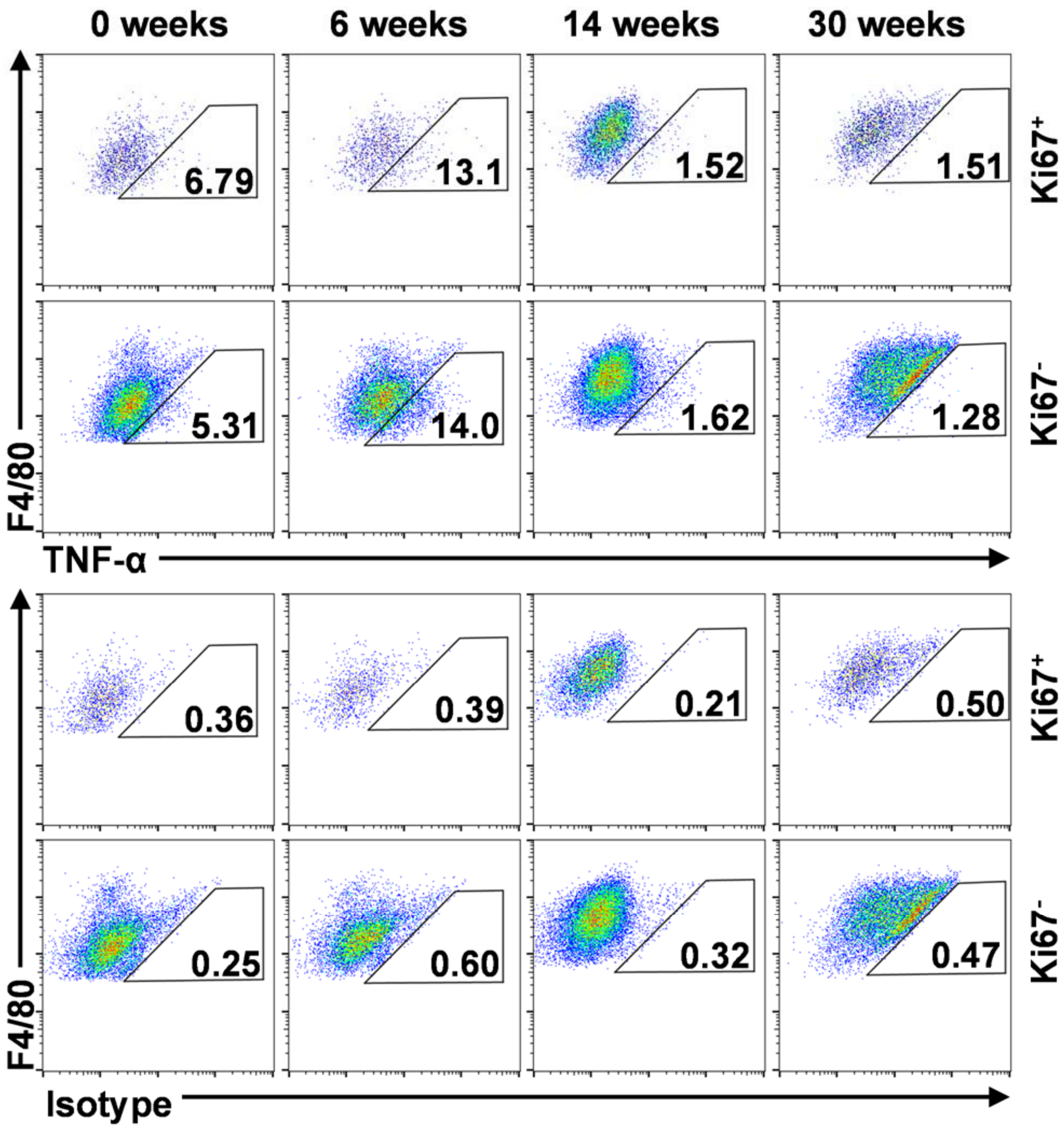

Figure S3. TNF-α expression in adipose tissue macrophages. Mice were treated with normal diet or high fat-diet for indicated time and epididymal adipose tissue were isolated for the analysis of TNF-α expression by intracellular staining.
